# Enhanced Water Solubility and Anti-Tumor Activity of Oleanolic Acid through Chemical Structure Modification

**DOI:** 10.3390/ijms232113291

**Published:** 2022-10-31

**Authors:** Zhicheng Gu, Shuxian Lin, Wanli Yan, Di Chen, Ziwei Zeng, Lei Chen, Yan Li, Bin He

**Affiliations:** 1 State Key Laboratory of Functions and Applications of Medicinal Plants, Engineering Research Center for the Development and Application of Ethnic Medicine and TCM (Ministry of Education), Guizhou Provincial Key Laboratory of Pharmaceutics, School of Pharmacy, Guizhou Medical University, Guiyang 550004, China; 2 School of Basic Medical Science, Guizhou Medical University, Guiyang 550004, China

**Keywords:** pentacyclic triterpenoids, oleanolic acid, water solubility, anti-tumor

## Abstract

Cancer has been a major health problem in the world in the past decades. It is urgent to develop new, effective and safe drugs for the treatment of cancer. There are many pentacyclic triterpenoids with positive anti-tumor activity and safety in nature. Oleanolic acid (OA), as one of the pentacyclic triterpenoids, also has broad biological activities including liver protection, anti-inflammatory, hypoglycemic, antiviral and anti-tumor. Therefore, to investigate its anti-tumor activity and mechanism, many OA derivatives have been developed. Some derivatives are less toxic to normal hepatocytes, which may be due to the strong liver protection ability of OA. However, the poor water solubility of OA is one of the main reasons for the weak anti-tumor activity. It is reported that some OA derivatives could enhance solubility by chemically linking some hydrophilic groups to improve anti-tumor activity. This review not only summarizes the highly water-soluble OA derivatives that can improve anti-tumor activity reported in recent years, but also introduces their possible anti-tumor mechanisms.

## 1. Introduction

Cancer is the second leading cause of death in the world. Although great progress has been made in the treatment of cancer, unfortunately, cancer is still one of the major global health problems [1,2,3]. Breakthroughs in basic and clinical research have created the possibility to treat cancer by surgery, chemotherapy, radiotherapy, targeted therapy, and immunotherapy [4]. Among these, chemotherapy is a very successful option, especially for cancers with concurrent metastases [5]. Unfortunately, the most common side effects of chemotherapy drugs mainly include neutropenia, anemia, bleeding, other manifestations of myelosuppression, organ toxicity, hepatotoxicity, nephrotoxicity, and so on, because most chemotherapy drugs are cytotoxic agents without obvious selectivity [6]. The major challenge for anticancer drug discovery is to bring about a therapeutic approach capable of attenuating malignant cells without damaging normal cells [7]. Therefore, chemotherapy eagerly needs the discovery of new, effective, and safer chemo-drugs [8].

Plants have been used as a source of medicine by humans worldwide for more than thousands of years, and they are reliable sources to discover and develop highly efficient and low toxic clinic medicines for treating human diseases [9]. Many compounds with anti-tumor properties are derived from natural products of medicinal plants and have strong capabilities to treat cancer (e.g., paclitaxel, vinblastine and camptothecin). These natural compounds usually can reduce the toxicity and side effects of chemotherapy and thus possess higher safety [10].

**Figure 1:**
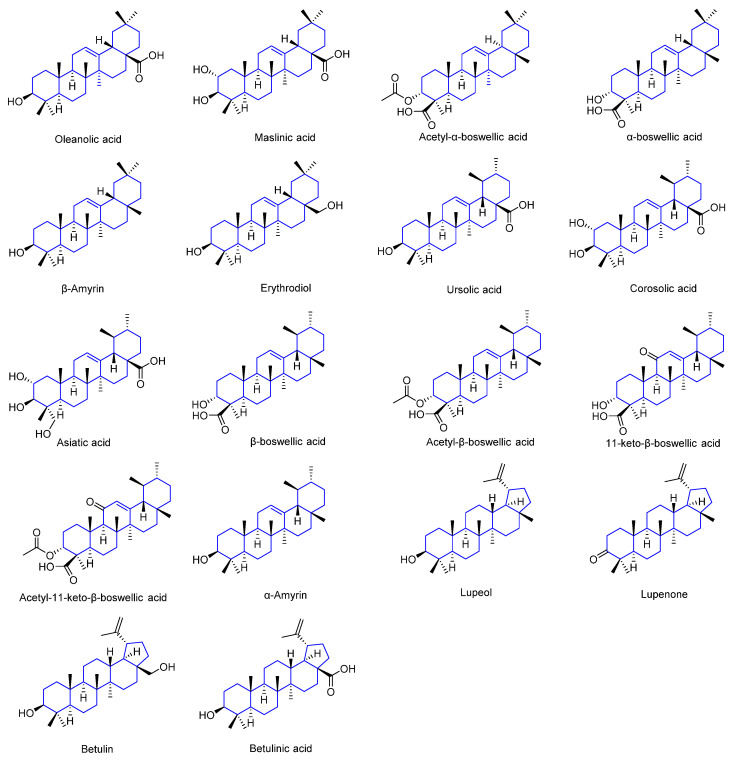
Structures of some biologically active pentacyclic triterpenoids.

**Figure 2:**
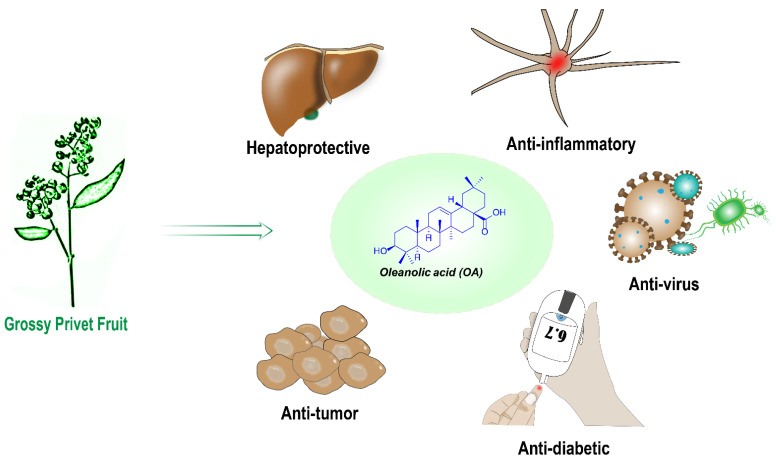
Biological activities of OA.

Among them, pentacyclic triterpenoids also come from natural products and have a variety of biological activities. Until now, more than 30,000 kinds of triterpenoids have been identified. A large number of pentacyclic triterpenoid derivatives have pharmacological activities; thus, they are widely studied and some are even used in clinical medicine [11,12,13]. Pentacyclic triterpenes are mainly obtained from various plants, such as soybean, alfalfa, birch, ginseng, olive pomace, mistletoe sprouts, clove flower, apple pomace, rosemary leaves, and so on [14,15]. The structures of some biologically active pentacyclic triterpenoids are shown in Figure 1 [16,17,18,19,20,21,22]. The main biological activities of these compounds include liver protection, anti-inflammatory, anti-blood sugar, anti-blood lipid, anti-virus, anti-AIDS, anti-tumor, and so on [14,15,23,24,25,26,27]. Oleanolic acid (OA), as a pentacyclic triterpene compound, has strong liver protection, anti-inflammatory, anti-virus, anti-diabetes, anti-tumor and other activities (Figure 2) [28,29,30,31,32]. Although OA has demonstrated certain anti-tumor activity and safety, its poor water solubility is still the main obstacle to improve its efficacy and clinical application [33,34,35]. A great number of studies have shown that OA can inhibit the proliferation of many tumor cells by inducing apoptosis and differentiation of tumor cells [36]. However, the anti-tumor activity of OA is relatively weak, mainly due to its poor water solubility [37]. It can be seen that improving the water solubility of OA may be one of the means to enhance its anti-tumor activity.

Until now, researchers have conducted a lot of work to improve the anti-tumor activity of OA. Changing the chemical structure of OA is the most economical, green and fastest method. Therefore, many anti-tumor agents from OA are mainly focused on changing the water solubility of OA to improve its anti-tumor activity [38,39]. The progress of OA and its derivatives in anti-tumor research in recent years (2015–2022) has been summarized. These derivatives based on OA show that water solubility is improved by introducing hydrophilic groups at the C-28 or C-3 position of OA, thus promoting the significant improvement of anti-tumor activity. In addition, the possible molecular targets and related mechanisms of OA in tumor study re discussed, which will facilitate in the further development of novel anticancer drugs based on the scaffold of OA.

## 2. Anti-Tumor Effect of OA

**Figure 3:**
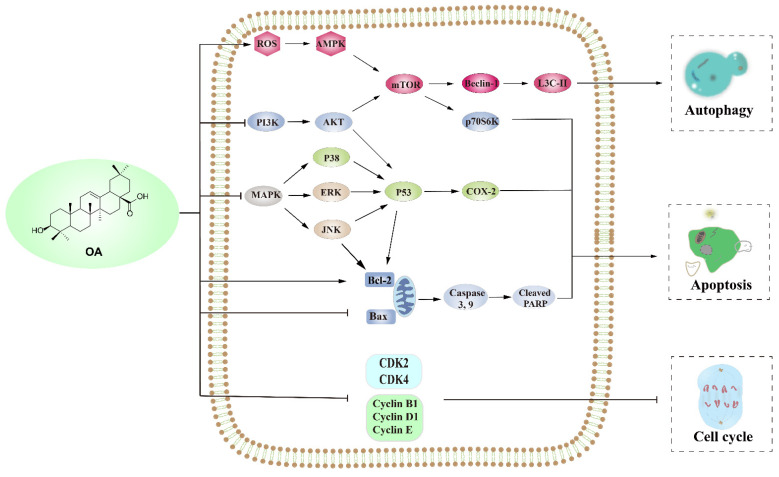
Three anti-tumor mechanisms of OA (activation “↑”, inhibition “T”).

OA can affect the growth of a variety of tumor cell lines including MCF-7 and MCF-7/ADR human breast cancer cells, 1321N1 astrocytoma cell line, hepatocellular carcinoma, HCT-116 colorectal cancer cells, etc. OA can control the growth of tumor cells through apoptosis, autophagy and the cycle of tumor cells (and its anti-tumor mechanism is far beyond these) [40]. The anti-tumor mechanism is briefly introduced (Figure 3).

### 2.1. OA Induces Apoptosis

Apoptosis acts as the body’s defense mechanism to remove damage, viral infections and cancer cells [41]. The structural changes in the DNA and cells in this process lead to nuclear and cytoplasmic condensation and cell disintegration into small membrane-bound structures [42]. OA can induce apoptosis of various tumor cells, such as human breast cancer MCF-7 cells, HepG2 cells, colon cancer HuH7 cells and other tumor cells [40].

#### 2.1.1. Upregulation Pathway of ERK/p53

Cyclooxygenase-2 (COX-2) is frequently expressed in different types of cancer. Many studies have shown that COX-2 promotes tumor cell apoptosis and participates in the apoptosis-inducing effect of apoptosis [43]. The activation of ERK leads to the upregulation of COX-2 in resveratrol-inducing apoptosis of ovarian cancer cells. Furthermore, OA can activate the expression of ERK and p53, thereby preventing apoptosis and cell proliferation, suggesting that p53 may play a key role in apoptosis upstream of OA [44]. OA can increase the expression of ERK and p53 to control the apoptosis pathway of cells through COX-2. Thus, the overexpression of COX-2 mediated by OA-activating ERK may be a possible mechanism for accelerating tumor cell apoptosis.

#### 2.1.2. Inducing Mitochondria-Mediated Apoptosis by Increasing the Bax/Bcl-2 Ratio

There are intrinsic and extrinsic pathways of apoptosis. The intrinsic pathway is regulated by mitochondria, and the extrinsic pathway is regulated by cell death receptors [45]. Bax/Bcl-2 ratio is a key factor in mitochondrial-mediated apoptosis. By inactivating anti-apoptotic members of the Bcl-2 family (Bcl-2, Bcl-xl, Bcl-w) such that cell survival can be inhibited, apoptosis could be increased. The upregulation of anti-apoptotic protein Bcl-2 and downregulation of pro-apoptotic proteins Bax can intrigue a mitochondrial dependent pathway [46]. The release of Cytochrome C can activate caspase-9, which then activates Caspase-3, Caspase-8 and other downstream caspases [47]. A study has reported that OA increased the expression of Bax and decreased the expression of Bcl-2 in SW-579 thyroid and PC-3 prostate cancer cells [48]. Western blot showed that the expression of Bax increased, and the expression of Bcl-2 and p-AKT decreased [49]. OA has been proven to induce apoptosis by downregulating the expression of Bcl-2 in many tumor cell lines, such as lung adenocarcinoma cells, HepG2 cells, MCF-7 and MDA MB-231 cells. OA controls cell apoptosis by increasing the Bax/Bcl-2 ratio and then releasing mitochondrial cytochrome C into the cytoplasm to initiate a caspase-dependent apoptosis [50,51].

#### 2.1.3. PI3K/AKT and MAPK Pathways

OA was found to regulate the expression levels of apoptosis-related and cell cycle-related proteins, as well as the activity of the PI3K/AKT pathway, in a dose-dependent manner [52]. The AKT-mTOR-p70S6K signaling pathway is considered to be a central regulatory pathway involved in regulating the expression of proteins involved in cell proliferation and survival. The PI3K/AKT and MAPK pathways regulate various cellular processes such as apoptosis, proliferation, metabolism and metastasis [44]. Some results have shown that AKT proteins were inhibited in AGS gastric cancer cells upon treatment with OA. The results indicated that OA might induce apoptosis by inhibiting the PI3K/AKT pathway in cancer cells [53].

Mammals have three major subfamilies of MAPK including ERK, JNK and p38. OA treatment significantly reduced the phosphorylation levels of AKT, ERK, JNK, p38 and p70S6K both in vitro and in vivo, while p53 phosphorylation was remarkably increased after OA treatment. The results suggest that OA profoundly modulates the activation of multiple CRC-related signaling pathways both in vitro and in vivo [54]. Novel insights showed that reactive oxygen species (ROS) and p53 signaling mediate p38 phosphorylation and caspase activation in colorectal cancer cell apoptosis. The cytotoxic activity of OA was consistent with the upregulation of p38, suggesting that the activation of p38 in apoptosis was induced by OA [44,55].

### 2.2. OA Induced Autophagy Death of Tumor Cells

Autophagy is a conservative self-degradation system, which is essential to maintain cellular homeostasis during stress conditions [56]. Autophagic cell death (type II programmed cell death) has become an important mechanism to induce cancer cell death through chemotherapy drugs and is becoming an attractive anticancer treatment [57].

#### 2.2.1. PI3K/AKT/mTOR Pathways

The PI3K/AKT/mTOR pathway plays an important role in coordinating biological processes such as cell proliferation, survival, and angiogenesis. Inhibition of this pathway is an important strategy for inducing apoptosis and inhibiting cell viability. The activation of PI3K leads to the sequential phosphorylation and activation of AKT and mTOR [58]. Once it is activated, AKT inhibits cell cycle arrest, stimulates angiogenesis and phosphorylates mTOR. The latter is involved in cell growth and survival, and regulates growth factors, cell nutrition and stress in response to cell signal transduction [59]. OA significantly inhibited the growth of HepG2 and SMC7721 cells by improving the formation of autophagic vesicles. At the same time, OA treatment also caused an increase in the distribution of microtubule-associated protein 1 light chain 3 (LC3) and the proportion of LC3-II and LC3-I. In addition, pretreatment with autophagy inhibitors 3-methyladenine (3-MA) and chloroquine (CQ) significantly rescued cell death induced by OA [60]. OA-induced autophagy is characterized by the formation of autophagic vesicles and acid vesicle organelles and the increase in autophagy-related proteins, which are regulated by the PI3K/AKT/mTOR pathway [61]. These studies have shown that OA depends on the PI3K/AKT/mTOR pathway to control autophagy.

#### 2.2.2. AMPK/mTOR Pathways

In addition to the PI3K/AKT/mTOR signaling pathway, the AMPK/mTOR signaling pathway is also one of the key pathways regulating autophagy [62]. Activation of AMPK increases the expression levels of Beclin-1 and LC3-II but decreases p-mTOR and p-ULK1 in tumor cells [63]. For HCT-116 cells and SW-480 cells, the expression levels of AMPK-α and p-mTOR were increased under the stimulation of 1 mM OA, which were checked by Western blot method. The data suggest that the anti-tumor cell growth activity of OA is closely related to the activation of the AMPK-mTOR signaling pathway. Activation of AMPK and inhibition of mTOR are two pathways of OA-induced autophagy and apoptosis. OA activation of AMPK is closely related to autophagy [64].

ROS mainly includes superoxide (O_2_^−^), hydroxyl radical (HO^−^) and hydrogen peroxide (H_2_O_2_), which are important signaling molecules leading to autophagic cell death. The study showed that the accumulation of ROS led to the inactivation of cysteine protease Atg4 and the accumulation of precursor of Atg8 ethanolamine phosphate. This is the key to starting autophagy formation, indicating that ROS may be the key to directly activate autophagy [65]. The results showed that OA could induce ROS accumulation in HepG2 cells in a concentration-dependent manner and induce the production of LC3-II in cells pretreated with the ROS scavenger, NAC. Therefore, OA may induce autophagic cell death in HepG2 cells by regulating ROS process. Therefore, it can be inferred that OA can induce apoptosis and autophagic cell death through an ROS-dependent pathway [60,66]. When the intracellular ROS level increases, AMPK is activated and thus leads to the increase in LC3-II [67].

### 2.3. Oleanolic Acid Regulates the Cell Cycle

Abnormal function of cell cycle regulators leads to uncontrolled cell proliferation, which makes them attractive therapeutic targets in cancer therapy [68]. By regulating the cell cycle of tumor cells, it is also one of the ways to regulate the growth of tumor cells.

Several studies have shown that OA can induce G1 cell cycle arrest in GBC-SD, NOZ, HCT15 and K562 cell lines. OA has been reported to induce S phase and G2/M phase cell cycle arrest in Panc28 and HepG2 cells in a concentration-dependent manner [44,69,70]. OA-treated U87 cells exhibited G1 arrest, in which increased p-ERK, p-JNK, p-AKT, p21, and p27 decreased Cyclin D1, CDK4, Cyclin E, and CDK2. Therefore, OA induces cell cycle arrest at different stages and then apoptosis in cancer cells. OA may alter the expression of cell cycle regulatory proteins differently in different types of cancer [40].

## 3. Anti-Tumor Study of OA Derivatives

**Figure 4:**
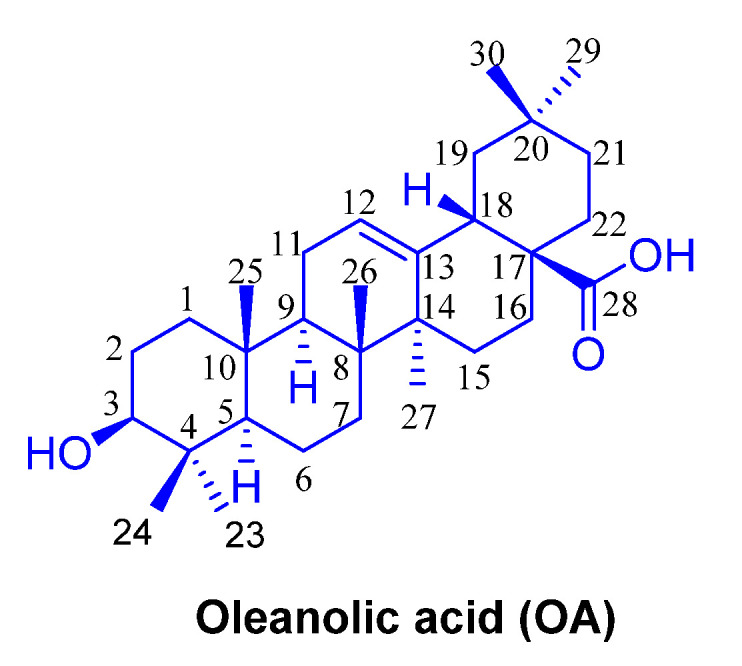
The chemical structure of OA.

As previously mentioned, due to the poor water solubility, the bioavailability of OA (Figure 4) is weak. To solve this issue, the research progress of OA derivatives in anti-tumor study has been made in recent years.

### 3.1. The Ionic Derivatives of OA

This section introduces a series of ionic derivatives based on the scaffold of OA. The design of such derivatives is mainly to improve the solubility of OA. Studies have shown that the mitochondrial membrane potential of tumor cells is higher than that of normal cells. Therefore, the increase in potential difference leads to the accumulation of cationic compounds with higher cytotoxicity and selectivity [71,72]. These derivatives mainly target mitochondria to regulate apoptosis of tumor cells.

**Figure 5:**
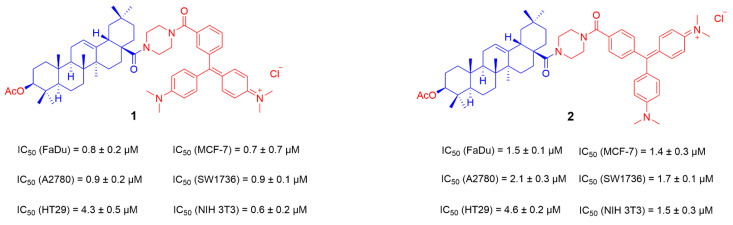
The chemical structures and anti-tumor activity of the ionic derivatives **1** and **2** of OA.

Compounds with cationic functional groups have attracted attention because cation transporters may facilitate their transport into cells to improve bioavailability [73]. Mitochondria is the power source of cells and the main intracellular organelles that maintain homeostasis, provide metabolic energy through oxidative phosphorylation, and regulate apoptosis. Mitochondrial dysfunction is closely related to cell death; therefore, designing drugs targeting cellular mitochondria has become an important strategy for tumor therapy [74,75]. Mitochondria in cancer cells is structurally and functionally different from healthy mitochondria, which contains higher mitochondrial membrane potential (MMP). Various lipophilic cations including TPP+, rhodamine, cyanamide cations, and cationic peptides attached to bioactive compounds will help them selectively accumulate in the mitochondria of cancer cells due to the higher MMP in cancer cells [76]. Recently, Sander Friedrich′s group has reported a series of cationic derivatives of OA to improve its biological activity and water solubility. The piperazinylamide as a linker was connected to the C-3 position-acetylated OA and meta- or para-substituted carboxylate malachite green to obtain compounds **1** and **2** (Figure 5). After the screening of the cytotoxic activity using the sulforhodamine B assay, both of them showed good anti-tumor activity in several human tumor cell lines (FaDu, A2780, HT29, MCF-7, SW1736 and NIH 3T3). The IC_50_ value of compound **1** in several tumor cells ranges from 0.7 to 4.3 μM, and the best value was achieved in MCF-7 cells (IC_50_ = 0.7 μM). Compound **1** obtained from para-substituted carboxylate malachite green was significantly more active than compound **2** obtained by meta-substituted carboxylate malachite green [77].

Previous research has shown that Rhodamine-123 can be accumulated in the mitochondria of SRC-transformed cells. These compounds showed certain selectivity toward cancer cells. In addition, a F-Rhodamine-docetaxel derivative showed high selectivity for targeting mitochondria and good anti-tumor activity [78]. Sven Sommerwerk and his team designed and synthesized triterpenoic acid derivatives by combining OA and Rhodamine B derivatives in 2017. The cytotoxicity of these derivatives was evaluated by using the SRB method in 518A2, A2780, HT29, MCF7, A549, 8505C and NIH 3T3 cells successively. Most of the compounds showed good biological activity against tumor cells (IC_50_ = 0.032–0.120 μM). Compound **3** showed good anti-proliferative activity in several tumor cell lines tested, especially for A2780 (IC_50_ = 0.032 ± 0.001 μM). It is less toxic to the non-tumor cell line NIH3T3 [79].

**Figure 6:**
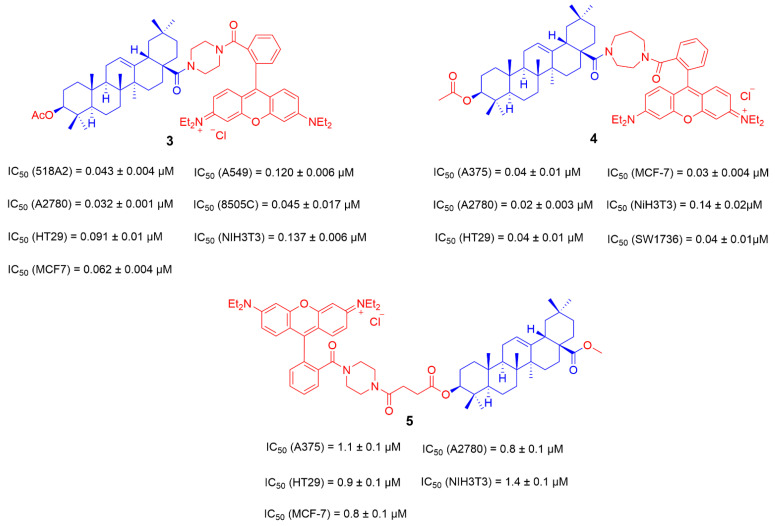
The chemical structures and anti-tumor activity of the ionic derivatives **3**–**5** of OA.

In 2018, Ratna Kancana Wolfram and his team continued to combine OA and Rhodamine B to synthesize OA derivatives by using the homopiperazinyl moiety to replace piperazine in compound **3** (Figure 6). The results showed that the cytotoxicity of these novel derivatives was comparable to that of previously prepared piperazinyl analogs. Among them, the anti-tumor activity of compound **4** (Figure 6) is slightly enhanced (IC_50_ (A2780) = 0.02 ± 0.003 μM) since these derivatives with good anti-tumor activity and selectivity can also target mitochondria [80]. In order to further improve their anti-tumor activity, a hybrid compound **5** (Figure 6) with the scaffold of Rhodamine B at the triterpene backbone C-3 was designed and synthesized by Niels Heise in 2021. However, compared with compounds **3** and **4**, the introduction of rhodamine B at C-3 of compound **5** cannot further improve the anti-tumor activity [81].

**Figure 7:**
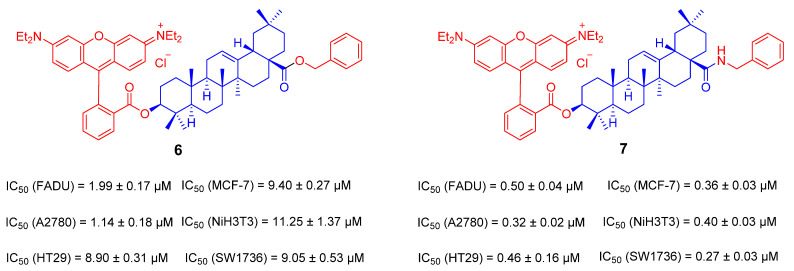
The chemical structures and anti-tumor activity of the ionic derivatives **6**, **7** of OA.

A series of cationic derivatives was obtained by directly coupling Rhodamine B and the C-3 position of OA and then introducing different substituents at the C-28 position. Then, their cytotoxicity was assessed. The results showed that the cytotoxicity of benzylamide compound **7** was stronger than that of benzyl ester derivative **6** (Figure 7). The IC_50_ of compound **7** in all tested tumor cell lines is less than 0.5 μΜ [81].

**Figure 8:**
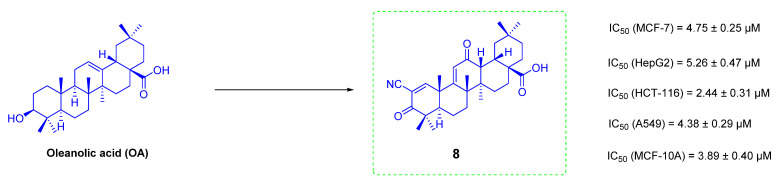
The chemical structures of OA derivative **8**.

Researchers have put a lot of effort into improving the anti-tumor activity of OA in the past few decades and have made great progress. The compound 2-cyano-3,12-dioxooleana-1,9(11)-dien-28-oic acid (CDDO, **8**, Figure 8) is one of the most excellent derivatives of OA (Figure 8). It has been developed for use in clinical studies in the treatment of melanoma, pancreatic cancer and kidney cancer [82]. However, the trial had to be terminated in clinical phase III due to some serious side effects. Triphenylphosphine cation (TPP+) is one of the most successful cationic lipophilic groups with several advantages including structural stability in biological systems, good solvent properties, and safety. It is widely used in the modification of anti-tumor drugs [83].

**Figure 9:**
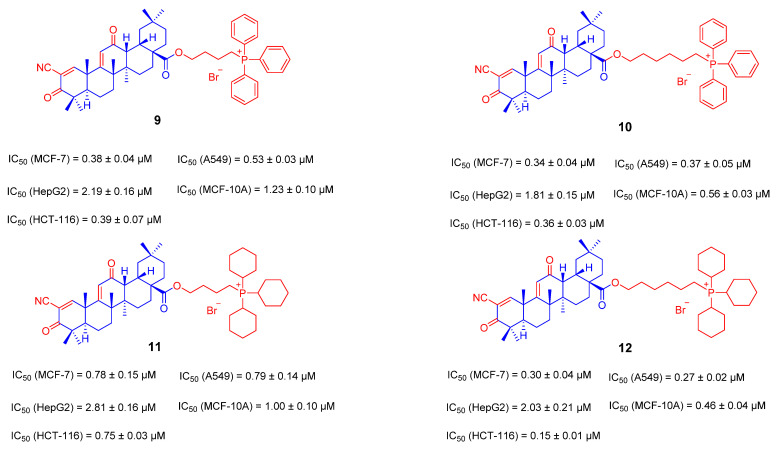
The chemical structures and anti-tumor activity of the ionic derivatives **9**–**12** of OA.

Recently, Wei Ju’s team reported a series of OA derivatives by introducing TPP+ or TCP+ moiety through alkyl chains of different lengths at 28-COOH of compound **8**. The linker length between OA and TPP+ had an effect on the activity, and the optimal length was n = 4, which is four methylene as a linker, as shown in compounds **9** and **10** (Figure 9). When the benzene ring in the triphenylphosphine cation (TPP+) was replaced by a saturated six-membered ring, the corresponding activity of compounds **11** and **12** (Figure 9) was improved less. Among these compounds, the inhibitory activity of TPP+ derivative **9** to tumor cells was higher than compound **8**, and its selectivity for tumor cells was also significantly improved. The experimental results further showed that the uptake of compound **9** was increased in the mitochondria of MCF-7 cells. It can cause a decrease in mitochondrial membrane potential and cell cycle arrest and induce apoptosis through mitochondria-mediated intrinsic pathways [84].

**Figure 10:**
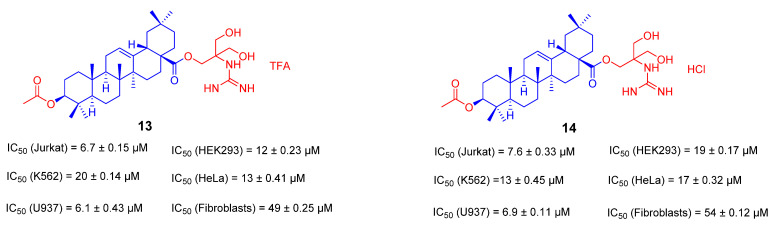
The chemical structures and anti-tumor activity of the ionic derivatives **13** and **14** of OA.

Considering that converting OA to ionic derivatives can improve bioavailability and selectivity, Anna Spivak’s group reported a novel cationic derivative of pentacyclic triterpenoids containing guanidino groups in 2018 [85]. Guanidine cations can enhance the efficient transport of biologically active substances across liposomes and cell membranes. In addition, the basic guanidino group (pKa = 13.5) can selectively deliver its anti-tumor molecules to tumor cells. At the same time, guanidine derivatives can be accumulated in the mitochondria of the scrotum to further induce tumor cell apoptosis. The derivatives **13** and **14** (Figure 10) were prepared as salts with trifluoroacetate or hydrochloride to improve the solubility and bioavailability of the derivatives. Finally, biological activity and selectivity were demonstrated. However, the best IC_50_ obtained in the U937 tumor cells was only about 6 μM [85].

**Figure 11:**
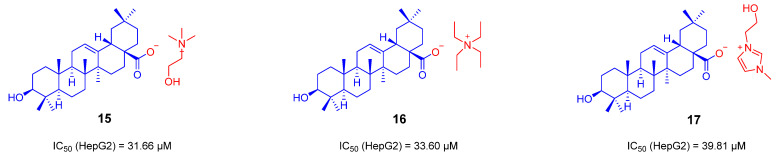
The chemical structures and anti-tumor activity of the ionic derivatives **15**–**17** of OA.

Recently, the Jie-Ping Fan’s group reported three ionic derivatives of OA [86]. Choline, tetraethylammonium and 1-(2-hydroxyethyl)-3-methylimidazole were selected as counter ions to assemble with OA to generate these ionic derivatives. They prepared ionic derivatives of OA by several simple syntheses and acid-base neutralizations, and finally converted them into simple organic salts. These ionic derivatives were characterized by HRMS, FTIR, NMR, XRPD, and TGA-DSC. The solubility of its derivatives was evaluated in the simulating gastric and intestinal fluids. The results showed that the solubility of OA is much lower than that of its ionic derivatives. The results of the apparent 1-octanol/water partition coefficient (Po/w) showed that the log Po/w of all ionic derivatives was lower than that of OA, indicating that the formation of ionic derivatives could improve their hydrophilicity compared to OA. The antiproliferative activity of the ionic derivatives on HepG2 cells was finally assessed by the MTT assay. The IC_50_ of the three ionic derivatives **15**, **16** and **17** (Figure 11) for HepG2 was 31.66, 33.60 and 39.81 μM, respectively, which were better than that of OA (IC_50_ = 80.73 μM) [86]. Again, these OA ionic derivatives control metabolic energy and cell apoptosis mainly by targeting cell mitochondria.

**Figure 12:**
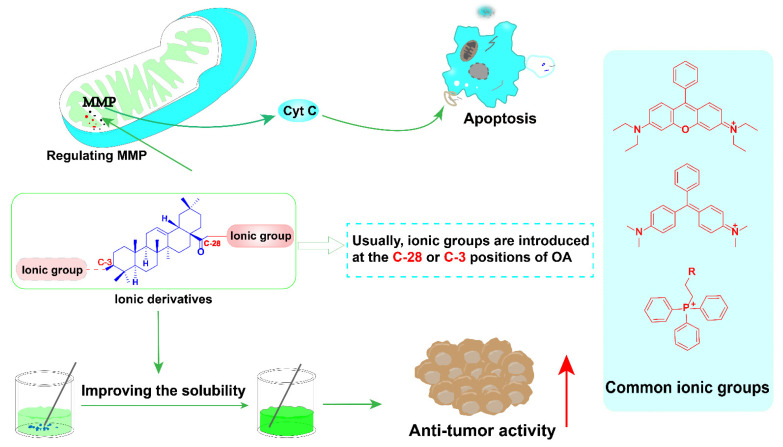
Structure activity summary of ionic derivatives of OA.

In a word, the above ionic derivatives can greatly improve the solubility of OA and increase the bioavailability of OA (Figure 12). Most of the cationic OA derivatives can change the permeability of the membrane by changing the mitochondrial membrane potential, increase the release of Cytochrome C to promote the apoptosis of tumor cells, and thus enhance the anti-tumor activity.

### 3.2. OA Saponin Derivatives

Saponins are amphiphilic molecules composed of carbohydrate and triterpene or steroidal ligand, which widely exist in nature, especially in many Chinese herbal medicines [87]. They have a variety of biological activities such as anti-fungal, anti-bacterial, anti-virus, anti-inflammatory, anti-oxidant, immunomodulation effects and anti-cancer. Triterpenoid saponins are a large group of natural products with structural diversity and biological activity diversity. In addition to natural saponins, chemical synthesis is also one of the effective methods to expand saponins [88,89].

Many of these saponins have N-acetylglucosamine in the carbohydrate fraction and are found to have significantly good water solubility and anti-tumor activity [90]. Although the poor water solubility of OA results in low oral bioavailability, simply modified OA with the introduction of hydrophobic groups cannot significantly improve its solubility [91]. Other studies have shown that the introduction of various glycosyl fragments into OA can improve water solubility and the corresponding biological activity [92]. This section summarizes a series of anti-tumor triterpene saponin derivatives designed and synthesized in recent years.

**Figure 13:**
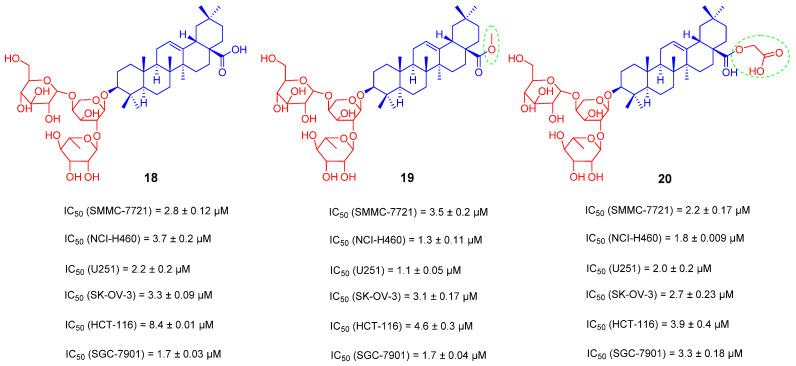
The chemical structures and anti-tumor activity of OA saponin derivatives **18**–**20**.

A series of triterpenoid saponins was isolated from pulsatilla chinensis (Bunge) regel, a traditional Chinese herb for ‘‘blood-cooling” and detoxification. Among these compounds, Hederacolchiside A1 (compound **18**, Figure 13), with a trisaccharide scaffold, manifests the strongest and broad-spectrum proliferation inhibitory activities (IC_50_ = 2.2–8.4 μM) against human cancer cell lines (SMMC-7721, NCI-H460, U251, SK-OV-3, HCT-116 and SGC-7901) [93]. However, the hemolytic toxicity prevented compound **18** from further in vivo studies. Fortunately, studies have disclosed that 28-COOH of triterpene saponins is the main functional group responsible for inducing hemolysis of erythrocytes [94]. In 2016, Yuanying Fang and his team have modified the carboxyl group of C-28 in compound **18**. The 28-COOH of compound **18** was replaced by a small fragment containing an ester group to obtain compounds **19** and **20** (Figure 13), which demonstrated strong anti-tumor proliferation activity in six tested cancer cell lines. In particular, compound **19** showed better antiproliferative activity against NCI-H460 and U2510 with the IC_50_ values of 1.3 and 1.1 μM, respectively. The safety of the compounds was also evaluated by the maximum tolerated dose in mice. The maximum tolerable dose of natural product **18** was less than 8 mg/kg, and 12 mice receiving 8 mg/kg died within 1 h. The maximum tolerable dose of compound **19** was greater than 168 mg/kg, and no mice upon this dosage of 168 mg/kg died within 7 days. The maximum tolerable dose of compound **20** was less than 168 mg/kg, and one mouse receiving 168 mg/kg died on day 7. Overall, the toxicity of compounds **19** and **20** was far less than that of the natural product **18**, suggesting that blocking the carboxyl group of C-28 in compound **18** could improve its antiproliferative activity and higher safety [93].

**Figure 14:**
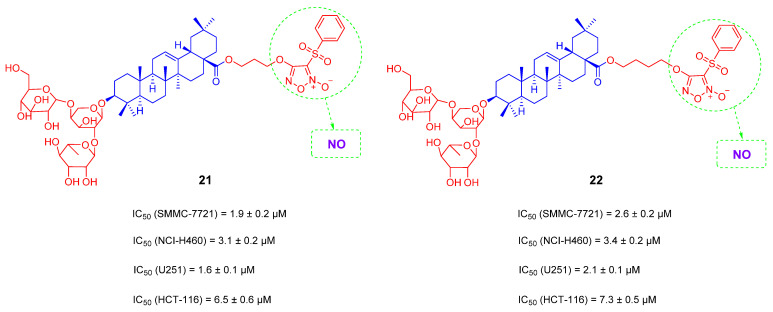
The chemical structures and anti-tumor activity of OA saponin derivatives **21** and **22**.

**Figure 15:**
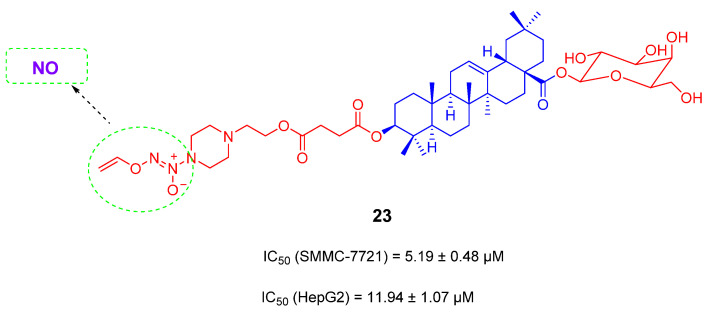
The chemical structures and anti-tumor activity of OA saponin derivatives **23**.

High concentration of nitrogen oxide (NO) can produce reactive nitrogen species, which along with reactive oxygen species can lead to deamination of DNA bases, cell function damage and apoptosis [95]. Furoxan fragment is an important NO-releasing group, which can produce high concentrations of NO to inhibit the growth of tumor cells [96]. In 2017, based on the lead compound **18** (Figure 13), Yuanying Fang’s team introduced the furan monomer fragment as a NO donor on 28-COOH of compound **8**. A series of anti-tumor derivatives was designed and synthesized. These derivatives containing NO donors can change the pharmacological activity and reduce the hemolytic toxicity. Among them, compound **21** (Figure 14) has the strongest capacity for NO release and shows certain cytotoxicity to four tested cancer cell lines (SMMC-7721, NCI-H460, U251, HCT-116). In particular, for U251, IC_50_ reaches 1.6 μM. Compound **21** was tolerated in mice at a dose of greater than 168 mg/kg, and no mice upon the dosage of 168 mg/kg died within 14 days. Compound **21** has a shorter linker than compound **22**, suggesting a longer linker may reduce the anti-tumor activity [97]. Zou-Yu and his team designed and synthesized compound **23** (Figure 15), which has improved 20-fold solubility than OA in aqueous solution [96].

**Figure 16:**
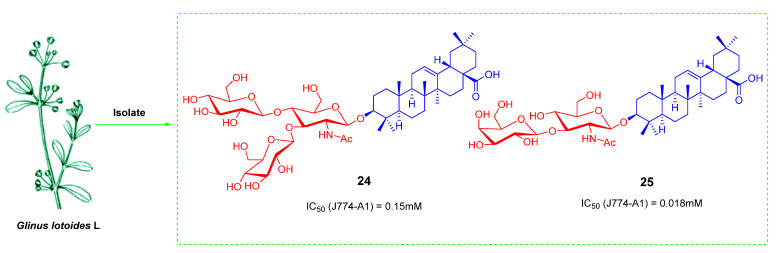
The chemical structures and anti-tumor activity of OA saponin derivatives **24**, **25**.

**Figure 17:**
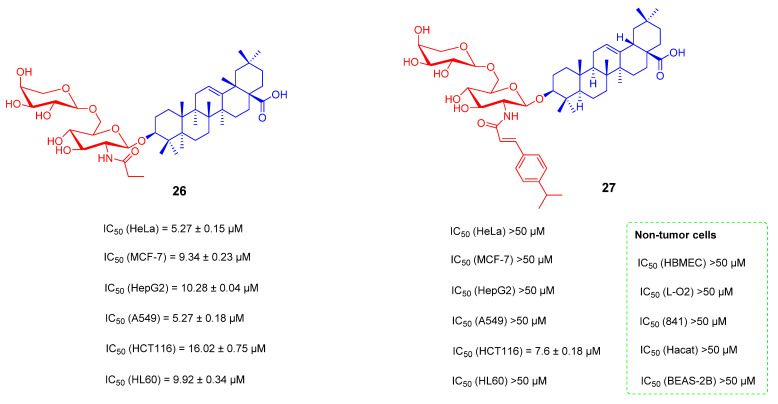
The chemical structures and anti-tumor activity of OA saponin derivatives **26**, **27**.

Triterpenoid saponins are natural products with structural diversity and biological activity diversity. These saponins contain N-acetylglucosamine in carbohydrates and have been found to have significant anti-cancer activity. For example, **24** (*lotoidoside*-D) and **25** (*Lotoidoside*-E) (Figure 16) were isolated from the root of *Glinus lotoides*-L, which had significant cytotoxicity in the J774-A1 cell line with IC_50_ values of 0.15 and 0.018 mM, respectively [98,99]. Previously, Huaping Zhang’s team reported the synthesis and evaluation of bioactivity of a natural oleanane triterpenoid saponin, Albiziabioside A, originally isolated from the aerial parts of Albizia inundata [100]. Gaofei Wei’s team designed and synthesized compound **27** (Figure 17) by coupling a substituent of a cinnamyl group at the scaffold of N-acetylglucosamine. The results showed that compound **27** had the best antiproliferative activity in HCT116 cells with an IC_50_ value of 7.6 ± 0.18 μM, but relatively weak cytotoxicity in other tumor cell lines including HeLa, MCF-7, HepG2, A549 and HL60 cell lines as well as in the normal cell lines. The antiproliferative activity of **27** may be related to the induction of cell cycle arrest and apoptosis in HCT116 cells with certain selectivity and safety [99].

**Figure 18:**
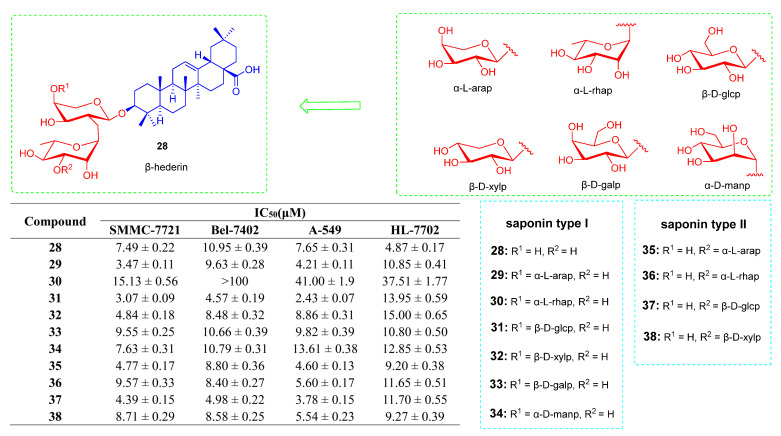
The chemical structures and anti-tumor activity of OA saponin derivatives **28**–**38**.

Liming Wang and his team reported a series of derivatives based on β-hederin and Hederacolchiside A1 that could inhibit the growth of tumor cells to some extent. A series of triterpenoid saponins based on β-hederin was synthesized by using linear or one-pot synthetic strategy of glycosylation (Figure 18) [101]. The synthetic saponin derivatives **28**–**38** (Figure 18**)** were evaluated for their anti-tumor effects on four cancer cell lines. Keeping in mind the IC_50_ values from 2.4 to 15.1 μM, the derivatives showed good antiproliferative effect against four cancer cell lines, except for compound **30**. As for saponins of the same type such as derivatives **29**–**34**, different compounds showed different cytotoxicity and selectivity against the tested cell lines. Different terminal monosaccharides affect antiproliferation activities against SMMC-7721 cells in the following order: **31** > **29** > **32**> **34**, **28** > **33** > **30** (Figure 18). The IC_50_ value of compound **31** of saponin type I against SMMC-7721 and A-549 cells reached 3.07 ± 0.09 and 2.43 ± 0.07 μM. Compound **37** of saponin type II showed good antiproliferation activity on A-549 cells (IC_50_ = 3.78 ± 0.15 μM) [101].

**Figure 19:**
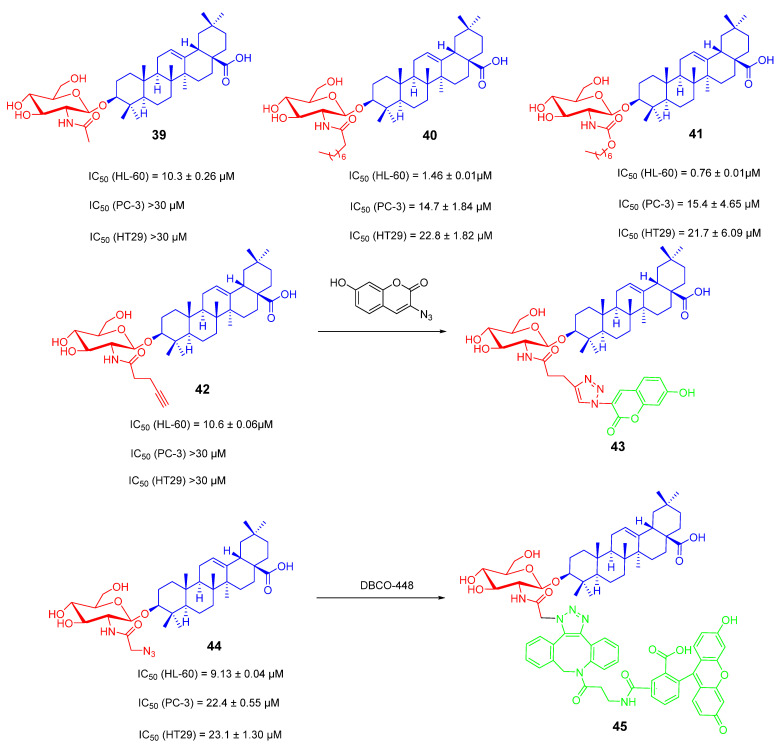
The chemical structures and anti-tumor activity of OA saponin derivatives **39**–**45**.

OA saponin derivatives containing N-acetylglucosamine have attracted much attention because of their good anti-tumor activity. However, the species of OA saponins containing N-acetylglucosamine structures are very rare in nature. Therefore, the diversity of their species containing N-acetylglucosamine can be expanded by chemical synthesis [102,103]. Compounds **40** and **41** (Figure 19) are amide and carbamate-based derivatives. They both exhibited stronger tumor cytotoxicity than compound **39** (Figure 19). Moreover, compounds **41** and **42** (Figure 19) are amide and carbamate-based derivatives exhibiting stronger tumor cytotoxicity than compound **40**. Additionally, compound **41** showed good selectivity and activity against the HL-60 tumor cell line with an IC_50_ value of 0.76 ± 0.01 μM. To explore the possible mechanism of this derivative, they designed and synthesized fluorescent probes **43** and **45** (Figure 19). After co-incubating the probes with cells, it was observed in the cellular uptake experiment that compounds **43** and **45** could penetrate into the cells and were mainly localized in the cytoplasm. The mitochondrial membrane potential detection experiment showed that compound **40** induced mitochondrial damage. The results are consistent with the distribution of compounds on the cytoplasm in the cell images. Therefore, the synthesized derivatives may induce apoptosis by interfering with mitochondria [104].

**Figure 20:**
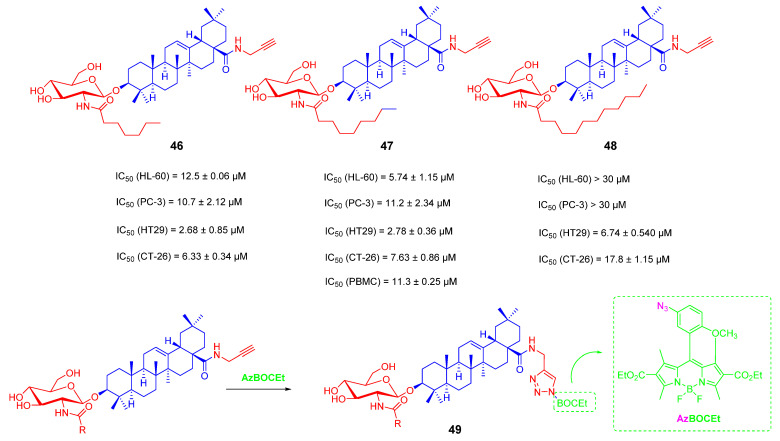
The chemical structures and anti-tumor activity of OA saponin derivatives **46**–**49**.

In 2020, Yu-Pu Juang and his team continued to report a series of N-acyl glucosamine-bearing saponin derivatives **46**–**49** (Figure 20) as anti-tumor studies. The main purpose of the research was to demonstrate the importance of carbon chain modification in cell distribution and cytotoxicity by changing alkanes with different carbon chain lengths on N-acyl glucosamine. The results showed that compounds **46** and **47** had comparable activity against HT29 cells with IC_50_ values of 2.68 and 2.78 μM, respectively. By introducing an alkynyl group at C-28 of OA saponins, the distribution of saponins was observed under a confocal microscope by the “click reaction” upon the catalytic copper ions to form the fluorescent probe **49**. The results showed that the saponin derivatives with a length of more than 12 carbons were mainly accumulated on the cell membrane, and their anti-tumor activity was greatly reduced. These derivatives with shorter carbon chains are more distributed into the cytoplasm. It was shown that the length of the carbon chain is an important factor to promote penetration into the cytoplasm. Therefore, this part of the saponin derivatives also exerts anti-tumor activity by penetrating into cells and then inducing apoptosis [105].

**Figure 21:**
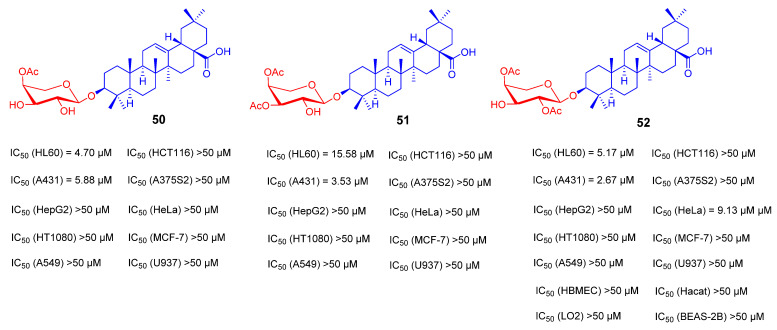
The chemical structures and anti-tumor activity of OA saponin derivatives **50**–**52**.

Studies have shown that the introduction of various glycosyl fragments can improve the anti-tumor activity of OA. Thus far, many reported triterpenoid saponins contain the α-*L*-arabinose moiety, such as β-hederin, Hederacolchiside A1 and Raddeanin A, which all show significant anti-tumor activity [106,107,108]. However, derivatives with a single substitute of α-*L*-arabinose at the 3-OH position have rarely been reported. Therefore, Ye Zhong’s team reported a series of derivatives **50**–**52** by coupling OA with a single α-*L*-arabinose (Figure 21). The derivative **52** was tested for in vitro anticancer activity against ten human cancer cell lines and four human normal cell lines by the MTT method. The results showed that compound **52** has significant selectivity and safety with IC_50_ values against the tested normal cells all above 50 μM. Furthermore, it showed good anti-tumor activity for A431 with an IC_50_ value of 2.67 μM. Preliminary study on the mechanism of the cell shows that these derivatives may induce cell cycle arrest and apoptosis through the mitochondrial pathway, and thus exert anti-tumor effects [109].

**Figure 22:**
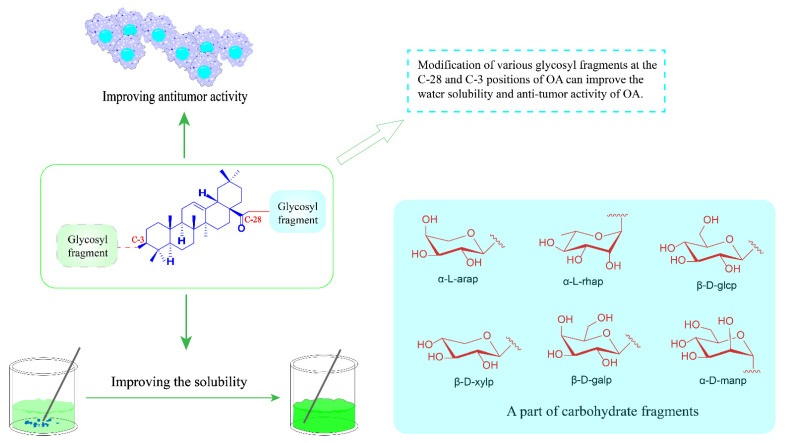
OA saponin derivatives improve the solubility.

The above OA saponin derivatives mainly introduce different glycosyl fragments at the C-3 or C-28 positions of OA by chemical means to enhance the anti-tumor activity and water solubility of OA (Figure 22).

### 3.3. Amino Long-Chain Derivatives of OA

In addition to OA saponin derivatives, scientists have made a lot of effort to the improvement of its water solubility by utilizing other strategies. For example, the cyclodextrins, micro-nanoemulsions, liposomes, polymeric nanoparticles and nanocapsules have been developed to improve the pharmacokinetic properties of these triterpenoids [110,111,112]. However, by chemically modifying the OA structure, it may be a greener and more economical way. In this regard, a series of amnio long-chain derivatives of OA have been developed. The bioavailability of OA is improved by introducing polar functional groups such as quaternary ammonium salts, glycosides, acylated oximes, amino acids or peptides, or acyl groups into the scaffold of OA by a semi-synthetic method [113,114]. This part mainly introduces the research status of these OA derivatives containing amino long-chain groups with anti-tumor properties.

**Figure 23:**
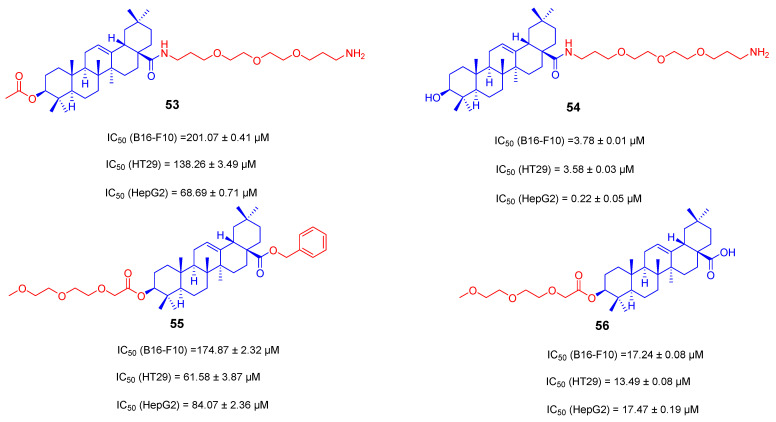
The chemical structures and anti-tumor activity of amino long-chain derivatives **53**–**56**.

Polyethylene glycol (PEG) polymers are considered as one of the multifunctional candidates for prodrug conjugation due to their excellent water solubility. Furthermore, covalent conjugation of polyethylene glycol to albumin can reduce the immunogenicity of albumin, and PEGylated biomolecules have longer blood circulation times than the corresponding unconjugated biomolecules [115]. In the work of Fatin Jannus’ team, they prepared several PEGylated compounds **53**–**56** that linked polyethylene glycol to the C-3 and C-28 positions of OA [116]. Three tumors cell lines of B16-F10, HT29 and HepG2 were used for the evaluation of their cytotoxicity by the MTT method. Compound **54** (Figure 23) showed high activity and selectivity against HepG2. Comparing compound **54** with **53** (Figure 23), any modification at the C-3 position of OA may prevent anti-tumor activity. Similarly, any modification at the C-28 position of OA may also hamper anti-tumor activity (**55** vs. **56**, Figure 23). Polyethylene glycol at the C-28 position, not at the C-3 position, may help to increase anti-tumor activity, if compared compound **54** with **55** (Figure 23). Compound **54** has an IC_50_ value of 0.22 ± 0.05 μM against HepG2 cells with a higher rate of early apoptosis. The PEGylated derivatives of these natural triterpenes can induce apoptosis in different tumor cell lines at low concentrations and may be novel and effective therapeutic drugs for the treatment of these diseases.

**Figure 24:**
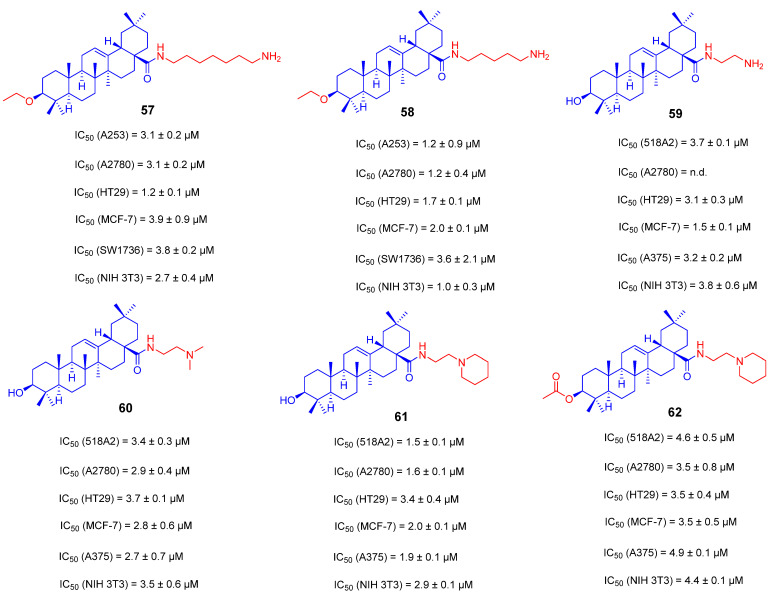
The chemical structures and anti-tumor activity of amino long-chain derivatives **57**–**62**.

In general, the introduction of the nitrogen atom into OA can improve water solubility by adjusting the acid–base balance. Based on an analysis of the *Comprehensive Medicinal Chemistry Database*, >25% of known drugs contain a carboxamide as a structural feature, suggesting that the synthesis of triterpenoic carboxamides might be helpful to find new cytotoxic agents for treating cancer [117]. Therefore, Michael Kahnt’s group used various α, ω-diamines and N-substituted ethylenediamines to design and synthesize a series of novel OA derivatives **57**–**62** (Figure 24). The cytotoxicity of these derivatives against different tumor cell lines was assessed by the photometric sulfur rhodamine B method. Among them, compound **58** has the most suitable length of carbon chain and better biological activity with IC_50_ values from 1.2 to 3.6 μM against A253, A2780, HT29, MCF-7, SW1736 and NIH 3T3 cells. The primary amine at the terminal was converted into a tertiary amine to obtain compounds **60**–**62**, which only demonstrated the comparable activity as that of compound **59** with a similar primary amine, suggesting that the C-28 position of OA modified either with primary amine or tertiary amine can improve anti-tumor activity [117].

**Figure 25:**
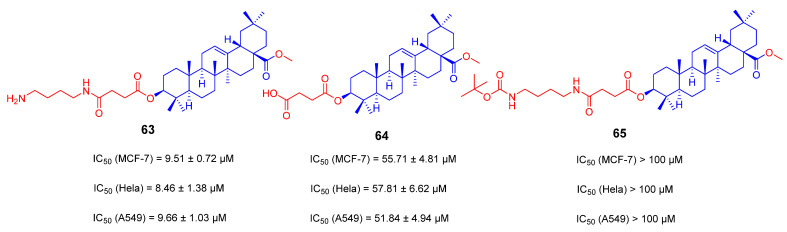
The chemical structures and anti-tumor activity of amino long-chain derivatives **63**–**65**.

Bin Feng, et al. have recently synthesized a series of pentacyclic triterpenoid derivatives **63**–**65** with ethylenediamine, butanediamine or hexamethylenediamine at the C-3 position of OA (Figure 25). Their antiproliferative activity was assessed against MCF-7, HeLa and A549 cells, respectively. Compared with the parent compound of OA, the anti-tumor activity of these derivatives was significantly enhanced. Among them, the IC_50_ value of compound **63** is 4.87 ± 0.77 μM against MCF-7 cells, suggesting that compounds with an amino group at the terminal may have superior activity to those with a carboxyl group or a shielded amino group, such as compounds **64** or **65** [118].

## 4. Conclusions

**Figure 26:**
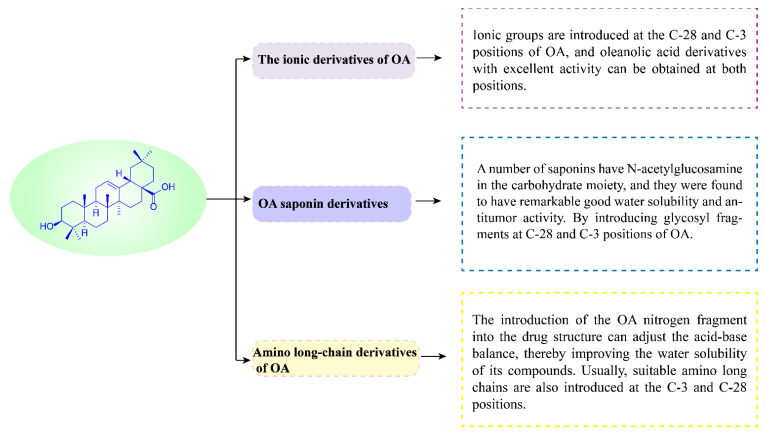
Three types of OA derivatives.

**Figure 27:**
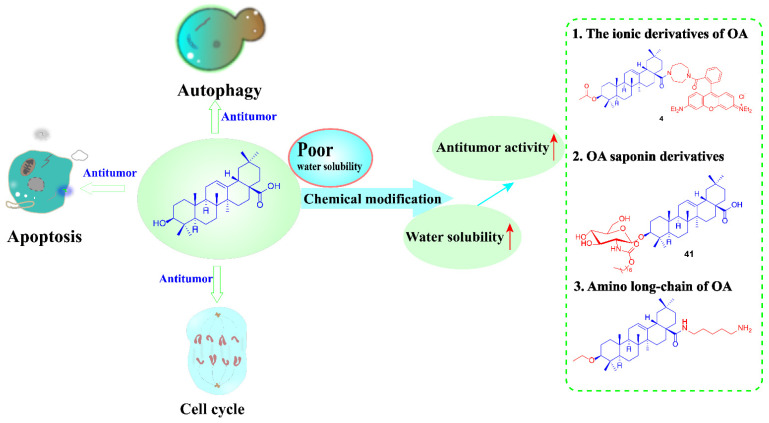
Anti-tumor mechanism of OA and representative compounds of three kinds of derivatives.

A large number of OA derivatives have been developed for anti-tumor study. The anti-tumor mechanism of OA and its derivatives, which mainly regulates apoptosis, autophagy and cycle of tumor cells, was extensively explored (Figure 26 and Figure 27). The weak biological activity of OA is mainly due to its poor water solubility. Therefore, enhancing the water solubility of OA is one of the means to enhance anti-tumor activity. Three kinds of hydrophilic groups were hybridized with OA to obtain OA derivatives with better anti-tumor activity (Figure 26). The first was ionic OA derivatives. This class of OA derivatives with cationic groups can specifically target mitochondria and then induce apoptosis in tumor cells since the mitochondrial membrane potential of tumor cells is higher than that of normal cells. Furthermore, ionic OA derivatives greatly improved water solubility and anti-tumor activity. The second type is saponin derivatives, which mainly introduce glycosyl fragments into OA and can greatly improve water solubility anti-tumor activity. The third type is long amino chains containing derivatives by changing the acid–base balance to increase water solubility. With the increase in water solubility, the anti-tumor activities of these three derivatives have also been significantly improved (Figure 27). Despite this progress, most researches are still in the primary stage, standing mainly in in vitro (or in cell) studies and rarely in in vivo animal studies. Furthermore, several novel strategies such as targeted protein degradation (e.g., proteolysis-targeting chimeras, PROTACs) have been utilized for modifying natural products to achieve more efficient therapeutic agents for drug development or molecular tools for mechanism study [119,120,121]. More detailed and in-depth research by using these novel strategies to develop novel OA derivatives is highly needed, which will facilitate in the further development of novel anticancer drugs based on the scaffold of OA.

## Data Availability

Not applicable.
